# Specific Binding and Endocytosis of Liposomes to HEK293T Cells via Myrisoylated Pre-S1 Peptide Bound to Sodium Taurocholate Cotransporting Polypeptide

**DOI:** 10.3390/vaccines10122050

**Published:** 2022-11-30

**Authors:** Shuji Hinuma, Kazuyo Fujita, Shun’ichi Kuroda

**Affiliations:** 1 Department of Biomolecular Science and Reaction, SANKEN, Osaka University, Mihogaoka 8-1, Osaka 567-0047, Japan; 2 Faculty of Human Life Science, Senri Kinran University, Fujisirodai 5-25-1, Suita, Osaka 565-0873, Japan

**Keywords:** hepatitis B virus (HBV), sodium cholate cotransporting peptide (NCP), liposome, myristoylated pre-S1 domain peptide, receptor binding, endocytosis, trypan blue, inhibitor

## Abstract

(1) Background: Sodium taurocholate cotransporting polypeptide (NTCP) functions as a key receptor for the hepatitis B virus (HBV) infection. Analyzing HBV and NTCP interaction is an important issue not only for basic research but also for the development of anti-HBV therapeutics. We developed here a novel model system to analyze the interaction of NTCP with liposomes instead of HBV. (2) Methods: Liposomal binding and endocytosis through NTCP in HEK293T cells were achieved by serial treatments of HEL293T cells transiently expressing NTCP-green fluorescence protein (GFP) fusion protein with a synthetic biotinylated pre-S1 peptide (Myr47-Bio) and streptavidin (SA) complex (i.e., Myr47-Bio+SA) followed by biotinylated liposomes. By this procedure, binding of [biotinylated liposomes]-[Myr47-Bio+SA]-[NTCP-GFP] was formed. (3) Results: Using this model system, we found that liposomal binding to NTCP on the cell surface via Myr47-Bio+SA was far more efficient than that to scavenger receptor class B type 1 (SR-B1). Furthermore, liposomes bound to cell surface NTCP via Myr47-Bio+SA were endocytosed into cells after cells were cultured at 37 °C. However, this endocytosis was suppressed by 4 °C or cytochalasin B treatment. (4) Conclusions: This model system will be useful for not only analyzing HBV entry mechanisms but also screening substances to prevent HBV infection.

## 1. Introduction

According to the World Health Organization, nearly 300 million people are estimated to be suffering from HBV infection worldwide in 2019. However, present therapeutic strategies are not necessarily enough to eradicate the diseases caused by HBV infection [1]. Understanding HBV infectious process in cells will be helpful not only as basic research but also to explore therapeutic and prevention tools against HBV infection. The early HBV infectious process in hepatic cells is currently presumed as follows [2,3,4]. At the initial step, HBV binds the cell surface. In the binding of HBV to the cell surface, NTCP (encoded by the *SLC10A1* gene) is convinced to play a crucial role as a receptor for HBV in the establishment of its infection. Subsequently, a complex of HBV and receptors are incorporated into cells. Then, the HBV genome or capsid is released into the cytoplasm. The N-terminal myristoylation of the pre-S1 domain of HBV surface large (L) protein is essential for HBV binding to NTCP and a synthetic myristoylated pre-S1 peptide (i.e., Myr47), which is a peptide mimetic of the pre-S1 domain of L protein, has been proven to have the ability to prevent HBV infection [5,6,7,8,9]. Myr47 (also referred to as Bulevirtide) is expected to be useful for the treatment of patients infected with hepatitis D virus (HDV)/HBV [10,11]. Therefore, the analysis of the HBV entry process is important in both aspects of understanding the HBV infection mechanism and the future development of prevention tools including anti-HBV drugs, antibodies, and vaccines. On the other hand, various molecules other than NTCP, such as heparan sulfate proteoglycan (HSPG), have been reported to exhibit the binding ability to HBV [2,3,4,12]. On the other hand, it appears that HBV can be endocytosed by a hepatic cell line, HepG2, independent of NTCP [13]. These facts raise the question of why HBV and NTCP interaction is indispensable in HBV infection and what is so different between NTCP and other receptors in the interaction with HBV.

So far, we have demonstrated that liposomes consisting of various phospholipids bind SR-B1 (encoded by the *SCARB1* gene) expressed on the cell surface and they are efficiently incorporated into HEK293T cells via SR-B1 [14,15,16]. Although SR-B1 is known as the primary receptor for high-density lipoprotein (HDL), it can bind not only HDL but also a variety of ligands, including liposomes [17]. These findings led us to examine the possibility that HBV binds SR-B1 as well as NTCP because HBV virions could be regarded as a sort of nanoparticles containing phospholipids. In the previous reports, we have demonstrated that both yeast-derived nanoparticles embedded with L protein, which is often referred to as bio-nanocapsules (BNCs), and Myr47 can bind SR-B1 expressed on the surface of HEK293T cells, and then they are endocytosed via SR-B1 [18,19]. These results strongly suggest that HBV particles can bind SR-B1 as well as NTCP. SR-B1 is widely expressed in various cell types including hepatic cells. We have shown that Myr47 can bind SR-B1, which is abundantly expressed on the surface of HepG2 cells, although they lack NTCP expression. HBV infection cannot be established in original HepG2 cells while the introduction of an NTCP expression plasmid makes HepG2 cells susceptible to HBV infection [20]. However, it is unclear whether there is any critical difference, which determines the susceptibility to HBV infection, in the interaction of HBV with NTCP different from the other receptors including SR-B1. To clarify this issue, it is necessary to precisely analyze the interaction between HBV and NTCP and the cellular events induced by their interaction. However, it is not always easy to chase the fate of HBV particles in cells because there are difficulties to conduct experiments using HBV particles, e.g., obtaining enough amounts of purified virions and keeping safety during experimentation. Therefore, the development of a simple and convenient model, which can reproduce an aspect of HBV and NTCP interaction, is required.

**Figure 1:**
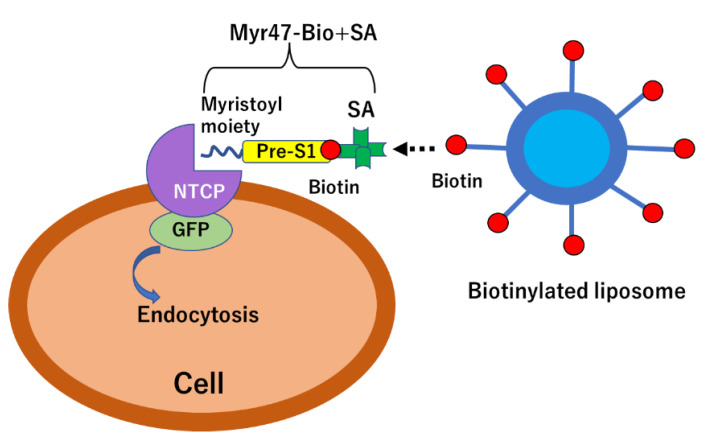
Illustration of liposomal binding and endocytic processes via Myr47 bound to NTCP-GFP expressed on the cell. In the 1st step, a Myr47+SA complex binds NTCP-GFP expressed on the cell. In the 2nd step, biotinylated liposome(s) bind this complex. In the 3rd step, an NTCP-GFP/Myr47-Bio+SA/biotinylated liposome(s) complex is endocytosed into the cell.

In the previous study, we conducted experiments using biotinylated Myr47 (Myr47-Bio) labeled with fluorescence-conjugated streptavidin (SA) and demonstrated that their complex can bind not only NTCP-GFP but also SR-B1-GFP expressed on HEK293T cells [20]. Based on these results, we developed here a unique method by which biotinylated liposomes bound a Myr47-Bio and SA complex (Myr47-Bio+SA), which was attached to NTCP-GFP on the surface of HEK293T cells in advance. As a single SA molecule has four biotin-binding sites, it can act as a linker to bind both Myr47-Bio and biotinylated liposomes (Figure 1). We show here that our model system using liposomes to mimic HBV and NTCP interaction is simple and useful to chase the binding of nanoparticles to NTCP on the cell surface via Myr47 and following their endocytosis. It was also possible to evaluate an inhibitory substance for NTCP-dependent entry of nanoparticles by utilizing this model system.

## 2. Materials and Methods

### 2.1. Preparation of Liposomes

We purchased 1,2-dioleoyl-*sn*-phosphatidylcholine (DOPC) from NOF Corporation (Catalog No.: COASTOME MC-8181; Tokyo, Japan) and biotinylated phospholipids, 1,2-distearoyl-sn-glycero-3-phosphoethanolamine-N-[biotinyl(polyethylene glycol)-2000] (DSPE-PEG-Biotin) and 1,2-dioleoyl-*sn*-glycero-3-phosphoethanolamine-N-(cap biotinyl) (PE-CAP-Biotin), from Avanti Polar Lipids (Alabaster, AL, USA), respectively. DiD (1,1′-dioctadecyl-3,3,3′,3′-tetramethylindodicarbocyanine, 4-chlorobenzenesulfonate) was obtained from Thermo Fisher Scientific (Waltham, MA, USA). Liposomes consisting of DOPC and various percentages (*w*/*w*) of biotinylated phospholipids, i.e., DOPC/DSPE-PEG-Biotin (0.01–10%) or DOPC/PE-CAP-Biotin (10%) were prepared according to the methods described previously [14,15,16]. Briefly, a lipid film was formed using phospholipids dissolved in chloroform. To prepare fluorescence-labeled liposomes, 1% (*w*/*w*) DiD was mixed with phospholipids before forming a lipid film. Then, it was suspended in phosphate-buffered saline (PBS) by vortex followed by sonication. About the same size of liposomes was obtained by extruding the suspensions through a 100 nm pore size membrane. The resultant suspensions were dialyzed against PBS. They were finally filtered through a 0.22-µm membrane and used for experiments. Physicochemical properties of liposomes, i.e., particle sizes and polydispersity indices (PDIs), were analyzed using the Zetasizer Nano ZS (Malvern Panalytical, Malvern, UK) as follows. (i) DiD-labeled DOPC/DSPE-PEG-Biotin (10%) liposomes [DiD-DOPC/PEG-Bio (10%)]: particle size = 127.8 ± 6.25 nm; zeta potential = −10.6 ± 3.50; PDI = 0.287 ± 0.020; *n* = 3. (ii) DiD-labeled DOPC/DSPE-PEG-Biotin (5%) liposomes [DiD-DOPC/PEG-Bio (5%)]: particle size = 107.8 ± 6.76 nm; zeta potential = −9.8 ± 0.84; PDI = 0.292 ± 0.069; *n* = 3. (iii) DiD-labeled DOPC/ DSPE-PEG-Biotin (1%) liposomes [DiD-DOPC/PEG-Bio (1%)]: particle size = 90.5 ± 12.40 nm; zeta potential = −9.2 ± 1.79; PDI = 0.200 ± 0.043; *n* = 3. (iv) DiD-labeled DOPC/DSPE-PEG-Biotin (0.5%) liposomes [DiD-DOPC/PEG-Bio (0.5%)]: particle size = 94.5 ± 13.10 nm; zeta potential = −8.9 ± 0.60; PDI = 0.301 ± 0.027; *n* = 3. (v) DiD-labeled DOPC/DSPE-PEG-Biotin (0.1%) liposomes [DiD-DOPC/PEG-Bio (0.1%)] liposomes: particle size = 77.4 ± 1.85 nm; zeta potential = −9.6 ± 1.20; PDI = 0.340 ± 0.044; *n* = 3. (vi) DiD-labeled DOPC/PE-CAP-Biotin (10%) liposomes [DiD-DOPC/CAP-Bio (10%)]: particle size = 81.1 ± 6.22 nm; zeta potential = −19.2 ± 2.03; PDI = 0.276 ± 0.017; *n* = 3.

### 2.2. Biotinylated Pre-S1 Peptides and SA

Myr47-Bio (Myr-GTNLSVPNPLGFFPDHQLDPAFGANSNNPDWDFNPNKDQWPEANQVK-biotin), biotinylated d11/13 (d11/13-Bio: Myr-GTNLSVPNPlGfFPDHQLDPAFGANSNNPDWDFNPNKDQWPEANQVK-biotin, in which d-amino acids were indicated in lowercase letters), and biotinylated aa2–48 (aa2-48-Bio: GTNLSVPNPLGFFPDHQLDPAFGANSNNPDWDFNPNKDQWPEANQVK-biotin), were obtained as described previously [19]. SA was purchased from Fujifilm Wako Pure Chemical Corporation (Catalogue No.: 198-17861; Osaka, Japan).

### 2.3. Cell Culture

HEK293T cells obtained from Riken RBC (Ibaraki, Japan) were grown in RPMI1640 medium supplemented with antibiotics and 10% heat-inactivated fetal bovine serum (BioWest, Bradenton, FL, USA) in a humidified atmosphere containing 5% CO_2_ at 37 °C as described previously [14,15,16,18,19]. HepG2 cells were maintained in the same medium as described previously [19].

### 2.4. Transient Expression of NTCP-GFP and SR-B1-GFP

NTCP-GFP and SR-B1-GFP expression plasmids were purchased from Sino Biological (Kanagawa, Japan). HEK293T cells were cultured in a 24-well plate and plasmid transfection was performed using PolyMagNeo (OZ Biosciences Inc., San Diego, CA, USA) as described previously [13,14,15,17,18]. HepG2 cells were cultured in a poly-L-lysine-coated 24-well plate and plasmid transfection was completed in the same manner as HEK293T cells.

### 2.5. Western Blot Analysis

We performed Western blot analysis as described previously [15,16,17,18,19]. Briefly, HEK293T cells transfected with or without an NTCP-GFP expression plasmid were cultured for 24 h, and then cell lysates were prepared using a RIPA buffer. These lysates were subjected to sodium dodecyl sulfate–polyacrylamide gel electrophoresis (SDS-PAGE) and then electrophoresed proteins were transferred to a PVDF membrane. After the membrane was treated with a blocking solution, we detected GFP on the membrane using a horse radish peroxidase (HRP)-conjugated mouse anti-GFP antibody (catalog number: 0518-34; Nacalai Tesque, Kyoto, Japan) at a dilution of 1:4000; antibody reaction was completed at 22 °C for 30 min. To detect the NTCP protein band, we employed a rabbit polyclonal NTCP (SLC10A1) antibody (catalog number: HPA042727; Sigma-Aldrich, St. Louis, MO, USA) as a primary antibody at a dilution of 1:1000; antibody reaction was completed at 4 °C for 24 h. An HRP-conjugated donkey polyclonal antibody against rabbit IgG (catalog number: A16035; ThermoFisher) was used as a secondary antibody at a dilution of 1:2000; antibody reaction was completed at 22 °C for 15 min. An HRP-conjugated mouse monoclonal anti-glyceraldehyde-3-phosphate dehydrogenase (GAPDH) antibody (catalog number: 015-25, 473; Fujifilm Wako Pure Chemical Co., Tokyo, Japan) was used at a dilution of 1:10,000; antibody reaction was completed at 22 °C for 30 min. To re-use the membrane, we stripped an antibody, which bound the filter once, using a WB stripping solution (Nacalai Tesque).

### 2.6. Liposomal Binding Assays Using Flow Cytometry

HEK293T or HepG2 cells transfected with or without plasmids in culture were harvested with trypsinization (cell membrane receptors were little affected by this treatment under our experimental conditions [14]). Cells were washed twice with PBS at 4 °C by centrifugation and then pelleted in a microtube. To prepare Myr47-Bio and SA complex which was referred here as to Myr47-Bio+SA, Myr47-Bio (200 nM) and SA (200 nM) were mixed in PBS in a microtube, and the mixture was left to stand at 22 °C for 30 min. Then, it was added to the cell pellets and the resultant cell suspensions were incubated on ice for 1 h. After cells were washed twice with cold PBS, DiD-labeled liposomes (DiD-liposomes) in PBS were added to them. After being incubated on ice for 1 h, they were washed twice with cold PBS. In flow cytometry, the fluorescence intensities (FIs) of 1−3 × 10^4^ cells for HEK293T cells and 3 × 10^4^ cells for HepG2 cells were analyzed, respectively, using a FACSCant™ II (BD Biosciences, Franklin Lakes, NJ, USA). We assessed the binding of DiD-liposomes to cells from a geometric mean of FI as described before [14,15,16,18,19]. Assays were completed in triplicate and data were expressed as means with standard errors. Statical analysis was performed using Student’s *t*-test.

### 2.7. Laser Scanning Microscopy (LSM)

The distribution of fluorescence-labeled liposomes in HEK293T cells transiently expressing NTCP-GFP or GFP was examined under LSM using FV1000 (Olympus, Tokyo, Japan) as described previously [14,15,16,18,19].

### 2.8. Quantification of Cells Endocytosing DiD-Liposomes Using Flow Cytometry and Trypan Blue

Liposomal endocytosis in HEK293T cells expressing NTCP-GFP was examined as follows. Cells, which were treated with Myr47-Bio+SA and DiD-DOPC/PEG(10%) as described above, were cultured in the culture medium (1 mL/well) in a 24-well microplate in a humidified atmosphere containing 5% CO_2_ at 37 °C for 1.5–3 h. After being harvested via pipetting, they were washed twice with PBS. Then, they were applied to flow cytometry. Trypan blue can quench fluorescence and it does not enter into live cells. Therefore, we used it to distinguish cell-surface bound and endocytosed DiD-liposomes. To detect cells endocytosing DiD-liposomes, we added 0.1% trypan blue (catalog No.: T8154; Sigma-Aldrich, St. Louis, MO, USA) in PBS to cells and performed flow cytometric analyses immediately to avoid long exposure of cells with trypan blue. In GFP-positive (GFP^+^) cell subsets, we assessed cell population endocytosing DiD-liposomes by gating in a two-parameter dot plot chart, in which gate was set to surround the area where fluorescence of cell-bound DiD-liposomes was quenched. Cells, which emerged in the gated area in the presence of trypan blue after they were cultured at 37 °C, were defined as cell population endocytosing DiD-liposomes. Cytochalasin B was purchased from Sigma-Aldrich (catalog No.: C2743). Assays were completed in triplicate and data were expressed as means with standard errors.

### 2.9. Actin Staining in HEK293T Cells Using Rhodamine Phalloidin

HEK293 cells (2.5 × 10^5^/mL, 300 μL/well) were cultured for 24 h in a poly-L-lysine-coated 8-well chambered coverglass (Thermo Fisher Scientific). After cells in culture were treated with cytochalasin B for 3 h, their actin was stained using rhodamine phalloidin. Actin staining was performed according to the manufacturer’s instruction using an F-Actin Visualization Biochem Kit (Cytoskeleton. Inc., Denver, CO, USA). Distribution of rhodamine fluorescence in HEK293T cells was observed under LSM.

## 3. Results

### 3.1. Specific Binding of DiD-DOPC/PEG-Bio (10%) to NTCP-GFP via Myr47-Bio+SA

In the previous study, we demonstrated that Myr47-Bio, which was labeled with Alexa 647-conjugated SA, bound cell surface NTCP by using HEK293T cells expressing NTCP-GFP. In addition, we showed that a complex of Myr47-Bio and Alexa647-conjugated SA bound not only NTCP but also SR-B1 [19]. The results of the previous study led us to examine an idea whether liposomes containing biotinylated phospholipids would bind Myr47-Bio+SA attached NTCP in advance as shown in Figure 1. Because a single molecule of SA has four binding sites, it was expected that Myr47-Bio+SA should remain at three biotin-binding sites, although in the mixture of Myr47-Bio and SA at equal molar (200 nM), the two molecules would not always react on a one-to-one basis.

**Figure 2:**
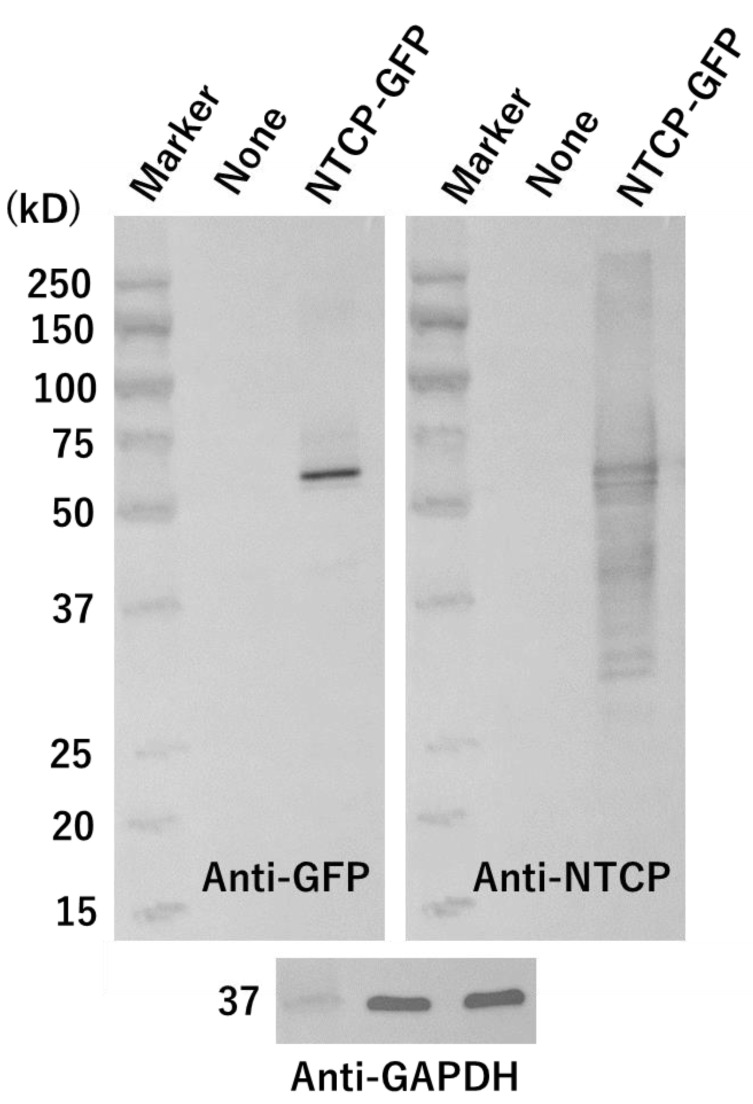
Western blot analysis of HEK293T cells transiently expressing NTCP-GFP. HEK293T cells transfected with (lanes indicated as NTCP-GFP) or without (those indicated as None) an NTCP-GFP expression plasmid were cultured for 24 h and then lysates were prepared from them. Cell lysates were subjected to SDS-PAGE at 2 μg of protein/lane under reducing conditions. The same membrane, to which electrophoresed proteins were transferred, was repeatedly used for the detection of target proteins by the indicated antibodies after an antibody bound to the filter was detached using a stripping solution.

**Figure 3:**
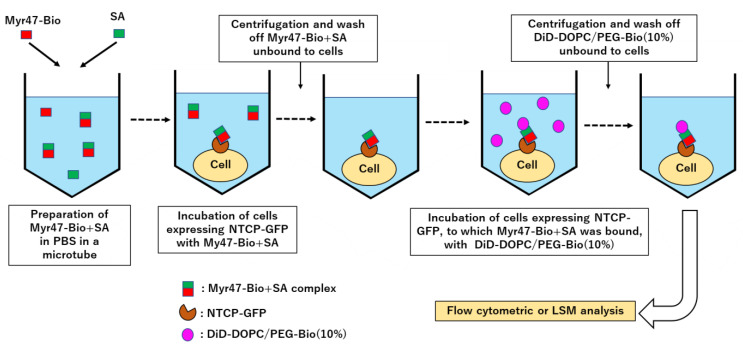
Schema of an experimental procedure to detect liposomal binding to NTCP-GFP expressed on HEK293T cells via Myr47-Bio+SA.

In this study, we transfected an NTCP-GFP expression plasmid into HEK293T cells. Using a Western blot, we confirmed that NTCP-GFP protein was expressed in these cells (Figure 2). The molecular weight of the NTCP-GFP fusion protein deduced from the amino acid sequence was about 65 kD. A major band was detected at the position of about 60–70 kD by both anti-GFP and anti-NTCP antibodies. These results indicated that intact NTCP-GFP protein was produced in HEK293T cells transfected with the expression plasmid. The schema of an experimental procedure to demonstrate liposomal binding to NTCP-GFP expressed on HEK293T cells via Myr47-Bio+SA is shown in Figure 3. We treated these cells with Myr47-Bio+SA and then washed off excess Myr47-Bio+SA. Subsequently, we examined the binding of DiD-liposomes containing biotinylated phospholipids, i.e., DiD-DOPC/PEG-Bio (10%), to Myr47-Bio+SA.

**Figure 4:**
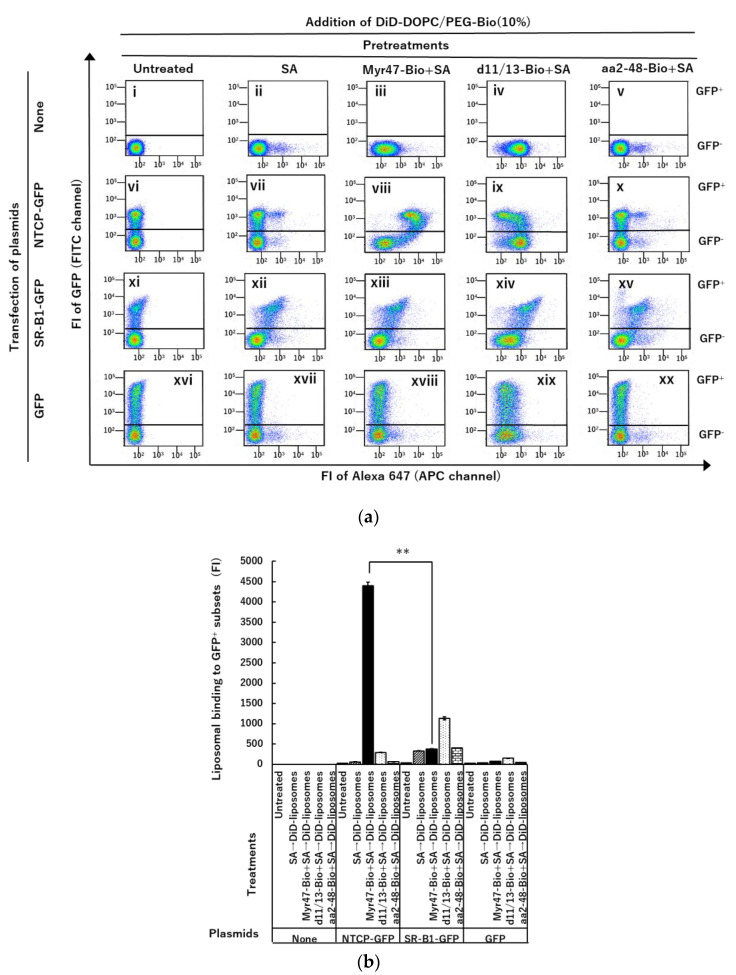
Flow cytometric analyses of liposomal binding to the surface of HEK293T cells transiently expressing NTCP-GFP via Myr47+SA. (**a**) Two-parameter dot plot analyses of liposomal binding to HEK293T cells expressing NTCP-GFP, SR-B1-GFP, or GFP. HEK293T cells transfected with or without plasmids indicated in the Figure were treated with SA, Myr47-Bio+SA (i.e., Myr47-Bio premixed with SA), d11/13-Bio+SA (i.e., d11/13-Bio premixed with SA), or aa2-48-Bio+SA (i.e., aa2-48-Bio premixed with SA) and then the binding of DiD-DOPC/PEG-Bio (10%) was examined. In panels (i~xx), upper and lower gates indicate GFP^+^ and GFP^−^ subsets, respectively. To obtain dot plot data, we analyzed 30,000 cells. (**b**) Binding of DiD- DOPC/PEG-Bio (10%) to GFP^+^ subsets of HEK293T cells with or without expressing NTCP-GFP, SR-B1-GFP, or GFP. FI of DiD-DOPC/PEG-Bio (10%) bound to the cell surface of GFP^+^ subsites was shown as a bar graph. Each data indicated in (**b**) corresponds to a GFP^+^ subset of a panel in (**a**). Data, which were obtained from 10, 000 in each assay, were expressed as geometric mean values ± standard errors (vertical bars) in triplicate assays. Statical analysis was performed using Student’s *t*-test; ** *p* < 0.01.

As shown in a two-parameter dot plot in panel (viii) of Figure 4a, DiD-liposomes specifically bound NTCP-GFP via Myr47-Bio+SA, that is, when the GFP^+^ subset was compared with the GFP-negative (GFP^−^) subset, the evident increase in liposomal binding was detected in HEK293T cells expressing NTCP-GFP after treatment with Myr47-Bio+SA. However, when the same cells were treated with d11/13-Bio or aa2-48-Bio, which were Myr47-Bio analogs not having the binding ability to NTCP, specific liposomal binding to NTCP-GFP was never detected (Figure 4a panels (ix) and (x), respectively). Although the binding of DiD-liposomes to the GFP^+^ subset of HEK293T cells expressing SR-B1 via Myr47-Bio+SA was detected (xiii), similar binding was observed in that via SA (xii). Therefore, specific liposomal binding to SR-B1-GFP via Myr47+SA was not evident. We have previously demonstrated that liposomes containing various phospholipids such as DOPC bind SR-B1 [15,16]. Therefore, at least a part of DiD-liposomes appeared to bind SR-B1-GFP independent of Myr47-Bio+SA. The profile of liposomal binding to Myr47-Bio+SA-treated HEK293T cells expressing NTCP-GFP well matched that of the fluorescence-labeled Myr47 observed in the previous study [19]. Interestingly, as shown in Figure 4b, the binding specificity to NTCP-GFP increased in the binding of DiD-liposomes via B-Myr47+SA more than that of fluorescence-labeled Myr47, which has been reported previously [19]. It should be also noted that DiD-liposomes bound more efficiently NTCP-GFP than SR-B1-GFP, which indicated that there is an obvious difference in the bindings of nanoparticles to NTCP and SR-B1 via Myr47 (Figure 4b).

**Figure 5:**
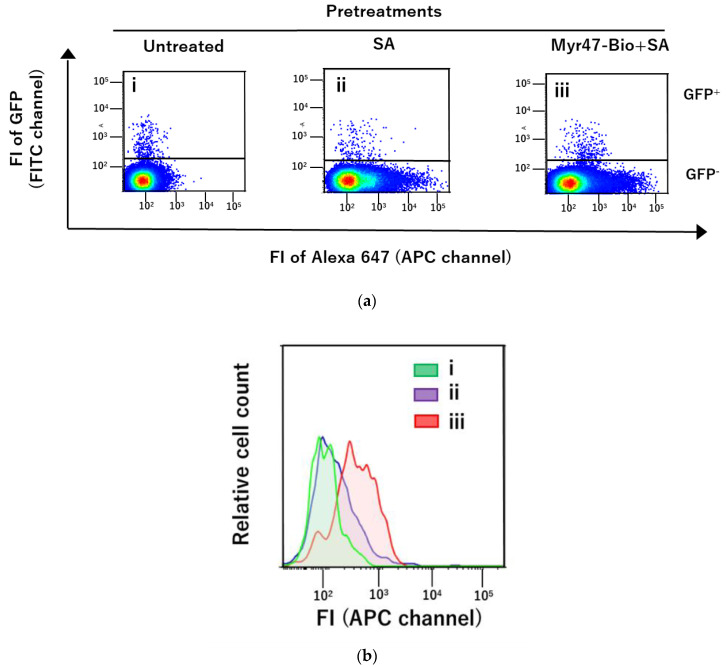
Flow cytometric analyses of liposomal binding to the surface of HepG2 cells transiently expressing NTCP-GFP via Myr47+SA. (**a**) Two-parameter dot plot analyses of liposomal binding to HepG2 cells expressing NTCP-GFP. The binding of DiD-DOPC/PEG-Bio (10%) to cells untreated (i), treated with SA (ii), or Myr47-Bio+SA (iii) was examined using flow cytometry. To obtain dot plot data, we analyzed 200,000 cells. As the GFP^+^ subset in HepG2 cells expressing NTCP-GFP was considerably fewer than that in HEK293T cells, dot plots were expressed using the large dot. (**b**) Histograms of GFP^+^ subsets, which bound DiD-DOPC/PEG-Bio (10%), in HepG2 cells expressing NTCP-GFP. Histograms (i~iii) indicated in the Figure correspond to GFP^+^ subsets of the dot plots (i~iii), respectively.

In this study, we used HEK293T cells to transiently express NTCP-GFP, because the transfection and protein expression efficacy of these cells were high when an NTCP-GFP expression plasmid was introduced into them. To know whether our approach can be applied to other cells as well as HEK293T cells, we transfected this plasmid into a hepatic cell line, HepG2, and examined whether the binding of DiD-DOPC/PEG-Bio (10%) to NTCP-GFP via Myr47-Bio+SA could be detected in HepG2 cells expressing NTCP-GFP. In these experiments, we employed a magnetofection method, because it little affected liposomal binding to the cell surface [15,16]. As shown in Figure 5, although the expression efficacy of NTCP-GFP in HepG2 cells was extremely lower than that in HEK293T cells (Figure 5a), specific liposomal binding to NTCP-GFP via Myr47-Bio+SA was detected in HepG2 cells (Figure 5b). These results indicated that our approach could be applied to HepG2 cells as well as HEK293T cells.

We also tested the binding of a premixed preparation of Myr47-Bio, SA, and DiD-DOPC/PEG-Bio (10%). However, it was difficult to see the specific binding to NTCP using such a premixed preparation containing liposomes, which indicates that the serial procedure, i.e., the first step of Myr47-Bio+SA attachment to NTCP and the second step of binding DiD-liposomes to Myr47-Bio+SA attached to NTCP, is crucial for achieving specific liposomal binding to NTCP via Myr47-Bio+SA.

**Figure 6:**
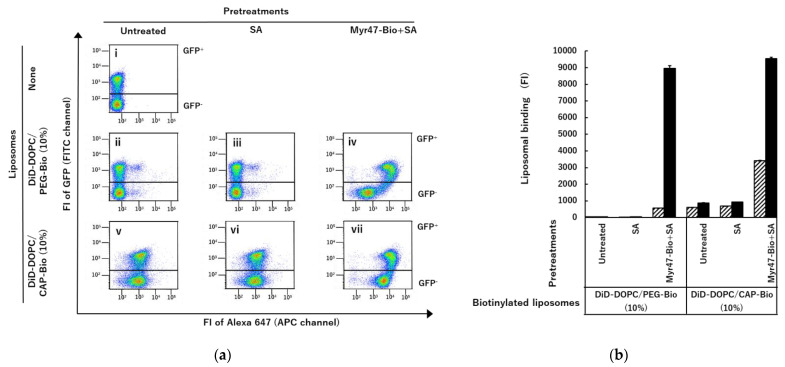
Differential binding of DOCP/PEG-Bio (10%) and DOPC/CAP-Bio (10%) to NTCP-GFP expressed on HEK293T cells via Myr47-Bio+SA. HEK293T cells transfected with an NTCP-GFP expression plasmid were pretreated with SA or Myr47-Bio+SA. Then, these cells were examined for the binding of the two biotinylated liposomes. (**a**) Two-parameter dot plots in flow cytometry; upper and lower gates indicate GFP^+^ and GFP^−^ subsets, respectively. To obtain dot plot data, we analyzed 30,000 cells. (**b**) Liposomal binding (FI) to GFP^−^ (striped bars) or GFP^+^ (black bars) subset was quantified in triplicate assays. Data were expressed as geometric means ± standard errors (vertical bars).

As shown above, our results indicated that liposomes containing biotinylated phospholipids were useful for obtaining specific binding of liposomes to NTCP via Myr47-Bio+SA. To examine whether the type of biotinylated phospholipids influences the binding specificity of liposomes, we compared binding patterns to HEK293T cells expressing NTCP-GFP between DiD-DOPC/PEG-Bio (10%) and DiD-DOPC/CAP-Bio (10%), which contain biotinylated phospholipids with a long and short spacer, i.e., (OCH_2_CH_2_)_45_ and (CH_2_)_5_, respectively, between a core phospholipid and a biotin moiety (Figure 6). DOPC/PEG-Bio (10%) and DiD-DOPC/CAP-Bio (10%) exhibited comparable binding to the GFP^+^ subset of HEK293T cells expressing NTCP-GFP as shown in Figure 6a panels (iv) and (vii), respectively. These results indicated that DiD-DOPC/CAP-Bio (10%) can bind NTCP-GFP similarly to DOPC/PEG-Bio (10%). However, in the absence of Myr47-Bio, DiD-DOPC/CAP-Bio (10%) (panel (v)) bound HEK293T cells expressing NTCP-GFP more efficiently than DOPC/PEG-Bio (10%) (panel (ii)). In addition, DiD-DOPC/CAP-Bio (10%) considerably bound the GFP^−^ subset (panel (vii)). These results indicated that DOPC/PEG-Bio (10%) is better to obtain specific liposomal binding to NTCP via Myr47-Bio+SA than DOPC/CAP-Bio (10%). We have previously demonstrated that liposomes consisting of DOPC bind SR-B1 endogenously expressed on HEK293T cells [16]. As quantification results were shown in Figure 6b, DOPC/CAP-Bio (10%) bound GFP^−^ subsets more efficiently than DOPC/PEG-Bio (10%). Therefore, DOPC/CAP-Bio (10%) would have a stronger interaction of its phospholipid part with SR-B1 than DOPC/PEG-Bio (10%).

**Figure 7:**
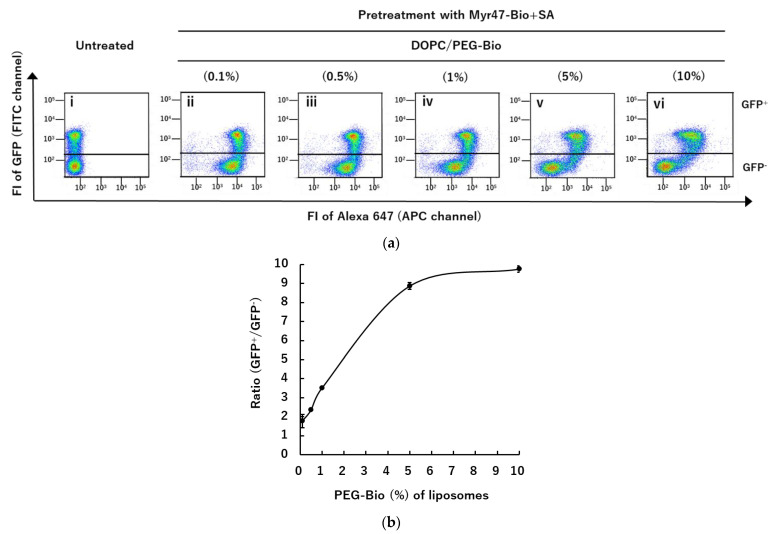
Contents of a biotinylated phospholipid required for specific binding of liposomes to NTCP via Myr47-Bio+SA. (**a**) HEK293T cells expressing NTCP-GFP were pretreated with Myr47-Bio+SA and then the binding of DOPC liposomes containing the indicated percentage of DSPE-PEG-Biotin (PEG-Bio) to these cells was analyzed using flow cytometry. To obtain dot plot data, we analyzed 30,000 cells. (**b**) The ratios of GFP^+^ to GFP^−^ subsets (FI) in the binding of DOPC liposomes containing indicated amounts of PEG-Bio are shown. Data were expressed as means ± standard errors (vertical bars) in triplicate assays.

In addition, we examined whether the contained amounts of biotinylated phospholipids (i.e., PEG-Bio) in liposomes influence the binding properties of liposomes to NTCP via Myr47-Bio+SA. As shown in Figure 7a panels (ii~vi), DOPC/PEG-Bio liposomes containing 0.1–10% PEG-Bio bound to NTCP-GFP^+^ subsets more efficiently than NTCP-GFP^−^ subsets. However, liposomes containing smaller amounts (0.1–1%) of PEG-Bio showed greater liposomal binding to NTCP-GFP^−^ subsets (panels (ii~iv)) than those containing 5–10% PEG-Bio (panels (v) and (vi)). The ratio, i.e., NTCP-GFP^+^/ NTCP-GFP^−^ subsets, of liposomal binding (FI) is shown in Figure 7b. An increase in the ratio reflects specific liposomal binding to NTCP. This ratio became larger as the contained amounts of PEG-Bio increased and it seemed to reach a plateau at 5–10% PEG-Bio. These results indicated that to obtain specific liposomal binding to NTCP via Myr47-Bio+SA, proper amounts of PEG-Bio (i.e., 5–10%) are necessary as a constituent of liposomes, although the ratio of GFP^+^/GFP^−^ using DOPC/PEG-Bio (10%) varied 10–15 among different experiments.

### 3.2. Liposomal Distribution in HEK293T Cells Expressing NTCP-GFP after Treatment with Myr47-Bio+SA Followed by DiD-DOPC/PEG-Bio (10%)

**Figure 8:**
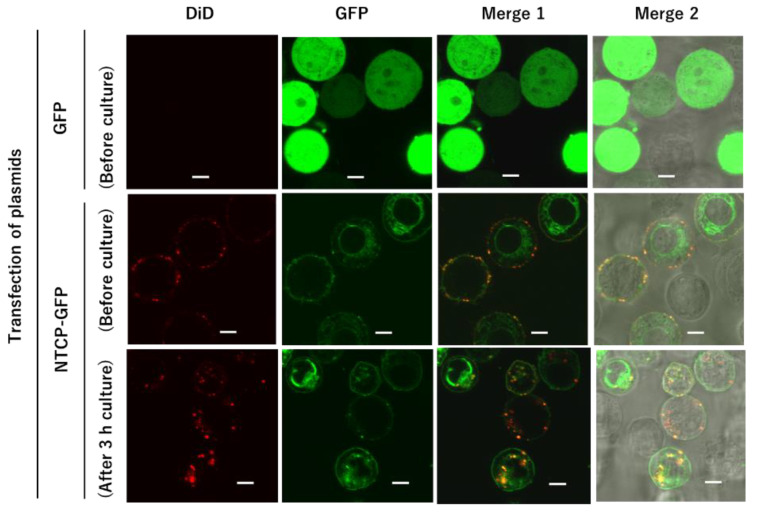
Cellular distribution of DiD-DOPC/PEG-Bio (10%) in HEK293T cells expressing GFP or NTCP-GFP after treatment with Myr47-Bio+SA followed by DiD-DOPC/PEG-Bio (10%). HEK293T cells transfected with GFP or NTCP-GFP expression plasmid were serially treated with Myr47-Bio+SA and DiD-DOPC/PEG-Bio (10%) on ice. These cells were washed with PBS and then a part of them was subjected to LSM as samples before culture (the upper and middle rows). The remaining cells were cultured in the culture medium at 37 °C, 5% CO_2_, for 3 h. After being washed with PBS, they were subjected to LSM (the lower row). DiD and GFP fluorescence detected under LSM are shown in red and green in the images, respectively. Merge 1: DiD and GFP images were merged and overlapping DiD and GFP fluorescence appeared to be yellow; Merge 2: Merge 1 and bright field images were overlaid; bars in the images indicate 5 μm.

We confirmed the cellular distribution of DiD-DOPC/PEG-Bio (10%) after HEK293T cells expressing NTCP-GFP were treated with Myr47-Bio+SA followed by DiD-DOPC/PEG-Bio (10%). Since these treatments were performed on ice, endocytosis could not happen. Subsequently, we examined whether these liposomes bound to the cell surface were endocytosed after cells were cultured at 37 °C. As shown in the middle row of Figure 8, cell surface localization of NTCP-GFP was observed in HEK293T cells expressing NTCP-GFP. In addition, co-localization of DiD-liposomes with NTCP-GFP was observed on the cell surface before culture (Merge 1 and 2 in the middle row of Figure 8). In contrast, GFP was broadly distributed in HEK293T cells expressing GFP. Liposomal binding to the cell surface of these cells was hardly detected under LSM (the upper row of Figure 8). These results were consistent with those of flow cytometric analyses in Figure 4 panel (xviii). After cells binding liposomes were cultured in the culture medium at 37 °C, in 5% CO_2_, for 3 h, liposomal internalization was observed in cells whereas cell surface liposomes appeared to decrease (the lower row of Figure 5). Liposomes and NTCP-GFP co-localized in cells (Merge 1 and 2 in the lower row), which suggested that a complex of liposomes, Myr47-Bio+SA, and NTCP-GFP was endocytosed into cells. These results indicated that liposomes bound to NTCP via Mry47-Bio+SA can be endocytosed into HEK293T cells under adequate conditions.

### 3.3. Quenching of DiD-Liposomes Bound on Cell Surface by Trypan Blue and Detection of Cells Endocytosing DiD-Liposomes in Flow Cytometry

By LSM analyses (Figure 8), we found that DiD-DOPC/PEG-Bio (10%) bound to NTCP-GFP via Myr47-Bio+SA was endocytosed into HEK293T cells after they were cultured. We subsequently tried to quantify this endocytosis using flow cytometry. To distinguish between cell surface-bound and endocytosed DiD-liposomes, we tested whether trypan blue can quench the fluorescence of DiD-liposomes binding to the cell surface. Trypan blue dye exclusion test is widely used to discriminate between viable and dead cells since this dye is not incorporated by viable cells. In addition, it is known to have a quenching ability against some fluorescence [21,22]. Therefore, we attempted whether trypan blue can quench DiD fluorescence of liposomes binding to the cell surface.

**Figure 9:**
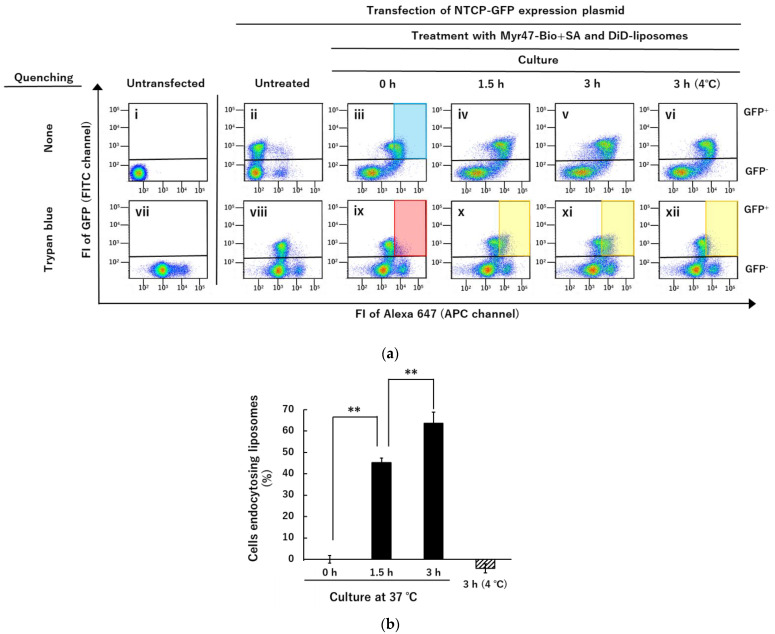
Detection of cell population endocytosing DiD-liposomes using flow cytometry and trypan blue. (**a**) HEK293T cells transfected with or without (untransfected) an NTCP-GFP expression plasmid were treated with or without (untreated) Myr47-Bio+SA followed by DiD-DOPC/PEG-Bio (10%). Subsequently, they were cultured for the indicated time. Then, they were subjected to flow cytometry. Upper and lower row panels indicate two-parameter dot plots obtained in the absence or presence of 0.1% trypan blue, respectively. The red area gated in panel (ix) was defined here as the GFP^+^ cell population whose fluorescence derived from DiD-liposomes bound to the cell surface was quenched by trypan blue. The blue area gated in panel (iii), which corresponds to the red area gated in panel (ix), represents the GFP^+^ cell population emitting the fluorescence of DiD-liposomes bound to the cell surface in the absence of trypan blue. The yellow gated area in panels (x~xii) corresponds to that in red in panel (ix). Cell culture was completed at 37 °C (iv/x) and (v/xi) or 4 °C (vi/xii). To obtain dot plot data, we analyzed 30,000 cells. (**b**) Cell populations in the gated areas in panels (ix~xii) were quantified and expressed as percentages which were calculated by the formula: (%) = [cell population in the gated area of the panel (ix~xii)—that of the panel (ix)]/[that of the panel (iii)—that of the panel (ix)] × 100. The assay was completed in triplicate and data were expressed as mean values ± SD (vertical bars). ** *p* < 0.01 in Student’s *t*-test.

As shown in panels (iii) and (ix) in Figure 9a, DiD fluorescence of liposomes bound to the cell surface appeared to be quenched by trypan blue because panel (ix) showed a similar pattern to panel (viii). These results indicated that at least a part of DiD fluorescence derived from liposomes binding to the cell surface NTCP-GFP could be quenched by trypan blue. Therefore, we expected that the fluorescence of endocytosed DiD-liposomes would become detectable in the red gated area set in panel (ix) even if in the presence of trypan blue because they would not be quenched by trypan blue. However, we should be careful of the following points: (1) because the background level of cellular fluorescence was considerably increased by the trypan blue treatment as shown in panels (vii) and (viii), the gated area defined in panel (ix) could not cover the whole GFP^+^ subset shown in panel (iii), i.e., the blue area gated in panel (iii) which corresponds to the red one gated in panel (ix), was estimated approximately 60% of GFP^+^ subset (this value varied 50–60% in different experiments); (2) as the pattern of two-parameter dot plots in panels (iv) and (v) seemed to be somewhat different from that in panel (iii), which suggested that DiD fluorescence could be altered by the internalization of liposomes. Although these points have to be taken into account, as we expected, after cells were cultured at 37 °C for 1.5–3 h, DiD-positive cells emerged in the areas gated in yellow in Figure 6a panels (x) and (xi), which were the same gated area shown in red in panel (ix). In contrast, as shown in panel (xii), in cells incubated at 4 °C for 3 h, DiD fluorescence-positive cells never emerged in the same gated area. These results indicated that at least a part of the cell population, which incorporated DiD-liposomes bound to the cell surface into cells, could be detected in the presence of trypan blue after cells were cultured at 37 °C.

As explained above, trypan blue treatment increased the basal FI of untransfected HEK293T cells and GFP^−^ subsets of those expressing NTCP-GFP (panels (vii) and (viii)). In this flow cytometric assay, we measured DiD fluorescence using an APC channel in which the excitation wavelength was 633 nm and a pass filter to detect emission was 650–670 nm wavelength. Our results suggested that trypan blue binding to the cell surface exhibits fluorescence detectable under these conditions, which seemed to be consistent with a report that trypan blue binding to the cell surface emits fluorescence by excitation light with a wide range of wavelengths [23].

Subsequently, we quantified the cell population that emerged in the gated area in GFP^+^ subsets (panels (x~xii)). Quantification results are shown in Figure 6b. Calculation of GFP^+^ cell population endocytosing DiD-liposomes (%) was completed using the following formula: (%) = [cell population in the yellow gate of the panels (x~xii)—that in the red gate of the panel (ix)]/[that in the blue gate of the panel (iii)—that in the red gate of the panel (ix)] × 100. The increases in fluorescent cells in the gated areas were detected time-dependently during culture for 1.5–3 h at 37 °C (Figure 9b). In contrast, in cells incubated at 4 °C for 3 h, such a fluorescence increase was never detected in the gated area (Figure 9b). These results strongly suggested that fluorescent cells emerging in the gated area represent at least a part of cells endocytosing DiD-liposomes. However, trypan blue treatment increased the basal level of cell fluorescence, which seemed to hamper the entire quantification of cells endocytosing DiD-liposomes: for example, the gated area in panel (iii) of Figure 9a could not include cells binding smaller amounts of DiD-liposomes. Therefore, further improvements will be required to distinguish cell surface-bound and endocytosed fluorescent liposomes in future studies. Nevertheless, considering the results of Figure 8 and Figure 9 together, these results indicated that DiD-liposomes bound to the cell surface NTCP via Myr47+SA are endocytosed into cells under adequate conditions.

### 3.4. Inhibition of Liposomal Endocytosis via NTCP and Myr47 by Cytochalasin B

**Figure 10:**
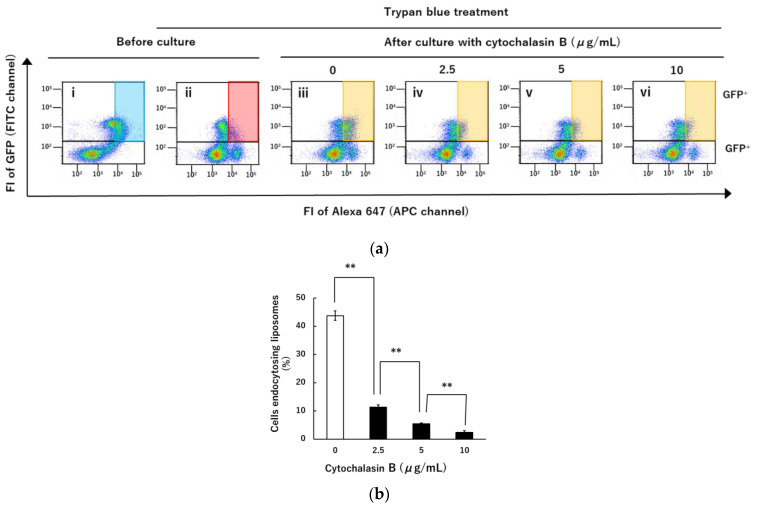

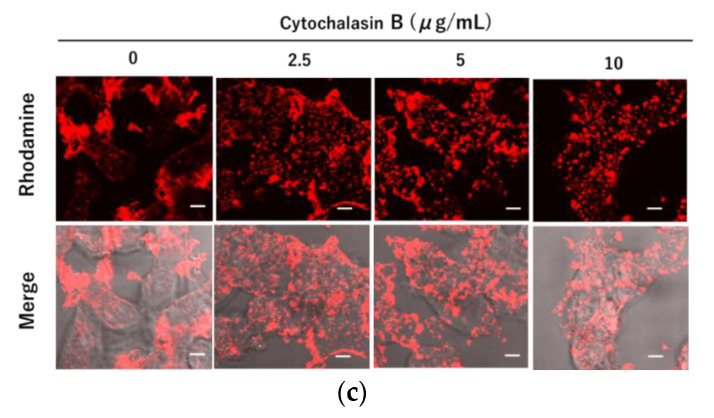
Inhibitory effect of cytochalasin B on internalization of DiD-liposomes bound to the cell surface. HEK293T cells transiently expressing NTCP-GFP were treated with Myr47-Bio+SA followed by DiD-DOPC/PEG-Bio (10%). After being cultured at 37 °C for 3 h in the presence or absence of cytochalasin B at the indicated concentrations, they were subjected to flow cytometry. (**a**) Two-parameter dot plot analyses of cells before and after they were cultured are shown in panels (i) and (ii) or (iii~vi), respectively. Analyses were performed in the absence (i) or presence (ii~vi) of 0.1% trypan blue. To detect cell population endocytosing DiD-liposomes, the gated area in red was set in panel (ii) in which fluorescence of cell surface-bound DiD-liposomes was quenched by trypan blue. The same gated area was applied to (i) and (iii~vi) too. To obtain dot plot data, we analyzed 30,000 cells. (**b**) Quantification of cell population emerging in the gated area after cells were cultured is shown in the bar graph. Cell population endocytosing DiD-liposomes were expressed as percentages which were calculated in this experiment as follows: [cell population in the gated area of the panel (iii~vi)—that of the panel (ii)]/[that of the panel (i)—that of the panel (ii)] × 100. The assay was completed in triplicate and data were expressed as mean ± SD (vertical bars). ** *p* < 0.01 in Student’s *t*-test. (**c**) Actin staining of HEK293T cells treated with cytochalasin B. To examine the effect of cytochalasin B on actin filament formation in HEK293T cells, they were cultured on the poly-L-lysine-coated cover glass. Treatment of cytochalasin B was performed at the same doses (2.5, 5, and 10 μg/mL) as those used in the experiments of panels (**a**,**b**) for 3 h. Actin was stained using rhodamine phalloidin. Rhodamine: rhodamine fluorescence is shown in red; Merge: rhodamine fluorescence and bright field images are merged. Bars indicate 5 μm.

We subsequently examined whether our model system using liposomes to mimic HBV and NTCP interaction could apply to evaluating endocytic inhibitors. Because the application of inhibitors is important for not only revealing cellular mechanisms of NTCP-dependent endocytosis but also considering the future investigation of anti-HBV entry substances such as low molecular weight compounds, peptides, and antibodies. To measure endocytosis of DiD-DOPC/PEG-Bio (10%) bound to the cell surface via Myr47+SA in HEK293T cells expressing NTCP-GFP, we used trypan blue because it could discriminate between cell surface-bound and endocytosed DiD-liposomes as described above. As shown in Figure 10a, the fluorescence of DiD-liposomes bound to the cell surface via Myr47-Bio+SA in the GFP^+^ population was detected in the absence of trypan blue (panel (i)) whereas it was quenched by trypan blue (panel (ii)). Therefore, we set the red area gated in panel (ii) to detect the GFP^+^ cell population endocytosing DiD-liposomes in the same manner as Figure 6a. After cells binding DiD-liposomes were cultured at 37 °C for 3 h, fluorescence-positive cells appeared in the gated area as shown in panel (iii) in Figure 7a. However, as shown in panels (iv~vi), the GFP^+^ cell population emerging in the gated area was reduced by the treatment with cytochalasin B which is known as a representative inhibitor for endocytosis [24]. Quantification of cell population endocytosing liposomes was expressed as percentages in Figure 10b. We found that endocytosis of liposomes was dose-dependently inhibited by cytochalasin B. As cytochalasin B is known to inhibit the multimerization of actin, we confirmed whether it affected actin filament formation in HEK293T cells under the experimental conditions employed here. As shown in Figure 10c, accumulation of actin filaments was observed in pseudopodia-like parts at the periphery of untreated HEK293T cells whereas small lumps of actin filaments were dispersed widely in cells treated with cytochalasin B. These results indicated that the actin cytoskeleton plays an important role in the process of NTCP/ Myr47-dependent liposomal endocytosis in HEK293T cells. We expect that the liposomal binding and endocytic system via NTCP/ Myr47 developed here can be applied to the evaluation of inhibitors for NTCP/Myr47-dependent endocytosis.

## 4. Discussion

We have recently demonstrated that Myr47-Bio+SA can bind not only NTCP but also SR-B1 using HEK293T cells expressing NTCP-GFP or SR-B1-GFP [19]. We expected that biotinylated liposomes would bind Myr47-Bio+SA, which has already bound NTCP or SR-B1, i.e., SA mediates the binding of biotinylated liposomes and Myr47-Bio as a linker. If it is possible, it could be a simple and convenient model system, which can mimic an aspect of the interaction between HBV and NTCP. In this system, Myr47-Bio is easily exchangeable with other biotinylated peptides. Thereby, we demonstrated that specific liposomal binding to NTCP happens via Myr47-Bio+SA using HEK293T cells expressing NTCP-GFP whereas it was not detectable when d11/13-Bio or aa-2-48-Bio was used instead of Myr47-Bio. With this approach, we compared the binding of DiD-liposomes to NTCP or SR-B1 via Myr47-Bio. Interestingly, liposomal binding via Myr47-Bio+SA to NTCP was 12 times more efficient than that to SR-B1. Our results were consistent with those of previous studies demonstrating a high-affinity receptor of NTCP for HBV [2,3]. Therefore, we believe that our approach developed in this study will be useful to reveal specific properties of the interaction between nanoparticles and NTCP. As to the HBV entry process, it has been reported the contribution of co-receptors such as HSPG and epidermal growth factor receptors [2,3]. Because our liposomal binding system is rather simple to analyze the interaction between nanoparticles and NTCP, it will be interesting to examine the role of co-receptors and other matters related to the HBV entry using our system in the future. The previous study indicated that the bindings of Myr47-Bio+SA to NTCP and SR-B1 were comparable levels [19]. However, as shown here, Myr47-Bio+SA-mediated liposomal binding to NTCP was considerably higher than that to SR-B1. Therefore, the binding manner of Myr47 to NTCP should be different from that of nanoparticles via Myr47 to NTCP. So far, PEGylated liposomes conjugated with Myr47 have been reported and these liposomes have been proven to provide excellent tropism for hepatocytes and the liver in vitro and in vivo, respectively [25]. However, their initial entry mechanism into cells including their interaction with NTCP has remained unclear. We believe that this is a report for the first time revealing the binding and endocytic processes of liposomes via NTCP and Myr47. In this study, we used HEK293T cells to express transiently NTCP-GFP because the efficacy of plasmid transfection was far higher in these cells than in the other cells such as HepG2. We expect that this model system can be applied to other cells. We showed here that our system can be applied to HepG2 cells too. However, it should be noted that to apply our approach to cells other than HEK293T cells, as a prerequisite, it will be required to ensure the high-level expression of NTCP-GFP in target cells.

To obtain specific binding of biotinylated liposomes to NTCP mediated by Myr47-Bio+SA, we investigated how to prepare liposomes containing biotinylated phospholipids. We found that liposomes containing 5–10% phospholipids modified with biotinylated PEG (i.e., DSPE-PEG-Biotin) are suitable to achieve specific liposomal binding to NTCP via Myr47-Bio+SA. We have reported that liposomes consisting of various phospholipids have binding properties to SR-B1 [15,16]. PEGylation of phospholipids appeared to hinder the interaction between phospholipids and SR-B1 endogenously expressed on HEK293T cells. As a result of reducing the binding of liposomes containing PEGylated phospholipids to SR-B1, liposomal binding specificity to NTCP via Myr47-Bio+SA appeared to increase.

Based on analyses using LSM and flow cytometry, we demonstrated that liposomes bound to the cell surface NTCP were internalized into cells after they were cultured at 37 °C. In flow cytometric analyses, we tried to distinguish between cell surface-bound and endocytosed DiD-liposomes using trypan blue. Trypan blue treatment quenched DiD fluorescence of liposomes bound on the cell surface in flow cytometry using the APC channel to detect DiD fluorescence, i.e., excitation and detective fluorescence wavelengths were 633 and 650–670 nm, respectively. Quenching efficacy was roughly estimated to be 80–90%, e.g., when populations in the gated areas were compared between panels (iii) and (ix) in Figure 9a. As fluorescence derived from DiD-liposomes bound to the cell surface was quenched at least partially by trypan blue, we could detect selectively fluorescence derived from endocytosed liposomes. However, trypan blue treatment of cells increased the basal levels of cell-derived fluorescence, which indicated that trypan blue bound to the cell surface emits fluorescence detectable within the range of 650–670 nm by 633 nm excitation light. This trypan blue-dependent cell-derived fluorescence, i.e., increasing the basal level of fluorescence, bothered the entire detection of endocytosed liposomes. Therefore, cells endocytosing small amounts of DiD-liposomes were unlikely detected by the trypan blue method employed here. Although there was such a limitation in the application of trypan blue to the detection of cells endocytosing DiD-liposomes, both results of LSM and flow cytometric analyses indicated that at least a part of DiD-liposomes bound on the cell surface NTCP was endocytosed into cells within several hours. In addition, we demonstrated that the endocytosis of DiD-liposomes bound on the cell surface NTCP was inhibited by cytochalasin B, which is an actin polymerization and endocytosis inhibitor [26]. We expect that the method developed in this study will be useful for not only the evaluation of endocytosis inhibitors but also the search for various molecules affecting NTCP-dependent entry of nanoparticles into cells.

By utilizing this NTCP interaction model system using liposomes instead of HBV, it became possible to analyze quantitatively the entry process of nanoparticles bound to NTCP by dividing the two steps of binding and internalization. Although we could clarify the high binding efficacy of liposomes to NTCP via Myr47-Bio+SA, there remain some questions to be answered regarding HBV entry into cells, e.g., detailed HBV internalization mechanism, the role of co-receptors such as HSPG in HBV entry, and the fate of HBV after incorporated into the endosome. Although further improvements will be required, we believe that our model system developed here has the potential to contribute to not only elucidating some of these basic issues related to HBV entry but also screening inhibitory substances for HBV infection.
